# Unintended socio-economic and health consequences of COVID-19 among slum dwellers in Kampala, Uganda

**DOI:** 10.1186/s12889-021-12453-6

**Published:** 2022-01-13

**Authors:** Rebecca Nuwematsiko, Maxencia Nabiryo, John Bosco Bomboka, Sarah Nalinya, David Musoke, Daniel Okello, Rhoda K. Wanyenze

**Affiliations:** 1 Department of Disease Control and Environmental Health, School of Public Health, Makerere University College of Health Sciences, Kampala, Uganda; 2 Kampala Capital City Authority, Kampala, Uganda

**Keywords:** Photovoice, Unintended consequences, COVID-19, Slum dwellers

## Abstract

**Background:**

To reduce the spread of COVID-19, several countries in Africa instituted countrywide lockdowns and other public health measures. Whereas lockdowns contributed to the control of the pandemic, there were concerns about the unintended consequences of these measures especially in the most vulnerable populations. We assessed unintended socio-economic and health consequences due to the COVID-19 pandemic and the mitigation measures among slum dwellers in Kampala to inform the on-going and future pandemic response strategies.

**Methods:**

This was a mixed methods cross-sectional study conducted in Bwaise I and Bwaise III slums of Kawempe division, Kampala Uganda from October to December 2020. We used systematic sampling to randomly select 425 household heads for the face-to-face quantitative interviews. We also conducted six focus group discussions (FGDs) with slum dwellers and used photovoice among eight Community Health Workers (CHWs) to document unintended socio-economic and health consequences. Quantitative data were imported into STATA version 14.0 for analysis, while qualitative data were analysed thematically using NVivo version 12. Modified Poisson regression analysis was conducted to establish factors associated with impact on access to food.

**Results:**

Most respondents reported limited access to food (71.1%; 302/425); disruption in education (77.1%; 270/350); drop in daily income and wages (86.1%; 329/382) and loss of employment (63.1; 125/198). Twenty five percent of the respondents (25.4%; 86/338) reported domestic violence as one of the challenges. Seven themes emerged from the qualitative findings on the impact of COVID-19 including: limited access to food; negative impact on children’s rights (child labour and teenage pregnancies) and education; poor housing and lack of accommodation; negative social behaviours; negative impact on family and child care; reduced income and employment; and negative impact on health and access to health care services.

**Conclusion:**

The slum dwellers of Bwaise I and Bwaise III experienced several negative socio-economic and health consequences of COVID-19 and its prevention measures that severely affected their wellbeing. Children experienced severe consequences such as child labour and teenage pregnancies among the girls. Response activities should be contextualised to different settings and protocols to protect the vulnerable groups in the community such as children and women should be developed and mainstreamed in response activities.

## Background

Coronavirus disease (COVID-19) has greatly affected the world leading to high morbidity and mortality [1]. To date (2nd December, 2021), there are 262,866,050 confirmed cases of COVID-19 and 5,224,519 deaths globally [2]. USA, India and Brazil were the most affected countries with over 48 million, 34 million and 22 million cases respectively. Africa has so far recorded over 8 million confirmed cases and 223,980 deaths. South Africa, Morocco and Tunisia were the most affected countries in Africa [3]. As of 2nd December, 2021, Uganda had a cumulative total of 127,618 confirmed cases of COVID-19 and 3252 deaths [2].

Frequent hand washing with soap or an alcohol based hand rub; use of face masks; maintaining physical distance; covering the mouth and nose when sneezing or coughing; and avoiding touching the mouth, eyes and nose with unwashed hands were the recommended and widely adopted individual prevention measures for COVID-19 globally [1]. To reduce community spread of COVID-19, several countries, Uganda inclusive, enforced physical distance, and instituted countrywide lockdowns that involved closing schools and international airports, restricted movement of people, and closure of workplaces, among other restrictions [4–6]. All these individual and community prevention measures were effective in reducing the number of COVID-19 infections though with severe consequences on people’s social, economic, health and psychological wellbeing [6–8].

In Uganda, the first case of COVID-19 was reported on March 21, 2020 [5]. Restrictions including a total lockdown were instituted on April 1, 2020 with closure of schools, public transport and formal workplaces except those offering essential services. In addition, the public was encouraged to practice hand hygiene, social/physical distancing and use of face masks in public spaces [5, 9]. However, there are concerns worldwide that most of the interventions have been “top-down” and may not have been appropriate to the local people in different contexts and likely led to negative socio-economic and health impact on the vulnerable groups in the communities [10, 11]. This has led to several unintended consequences and increased vulnerability in different populations [10]. A body of literature exists on how public health measures negatively impact communities [12, 13]. Unintended consequences normally result from poor policy design, unclear policy goals and inappropriate evidence use [12]. Identifying negative unintended consequences helps in mitigating the effects and planning for prevention of the same.

A study on the socio-economic vulnerability to COVID-19 in the Greater Kampala Metropolitan Area in Uganda showed slum dwellers as the most vulnerable group with a low adaptive capacity [14]. A similar study among urban refugees living in slums in Uganda revealed increased income insecurity, sexual and gender-based violence and anxiety during the lockdown [15]. The impact of COVID-19 among the local Ugandan slum dwellers is likely to be much more since they had no organised financial benefits from the government or international relief aid compared to the refugees. Slums in Kampala are characterized by overcrowding with many people living together and in close quarters which makes practicing social distancing, a critical prevention strategy in combating COVID-19, problematic. In addition, slums lack access to adequate basic services such as sanitation, water, food and basic health care services which further increases their vulnerability [16, 17]. This study assessed unintended socio-economic and health consequences due to the COVID-19 pandemic and the mitigation measures among slum dwellers in Kampala so as to draw lessons to inform the on-going and future pandemic response strategies.

## Methods

### Study setting

The study was conducted in Bwaise I and Bwaise III slums of Kawempe division, Kampala Uganda from October to December 2020. Bwaise is one of the largest slum areas in Kampala with a mixture of commercial, industrial and residential settlements. Bwaise I slum has a total population of 37,500 and an average household size of 5. Bwaise III has a population of 35,000 people and also an average household size of 5 [18]. The two slums are characterised by poor infrastructure and service provision which creates poor housing, flooding and challenges in access to water, sanitation and hygiene [16]. The two slums are located within the Kampala Central business District (CBD) which has a high COVID-19 exposure index [14]. According to a study on the socio-economic vulnerability to COVID-19 in the Greater Kampala Metropolitan Area, Bwaise I and Bwaise III slums had the lowest adaptive capacity. Adaptive capacity in that study was assessed in terms of food security, level of income and access to good health care [14]. We conducted the study between October and December 2020, after the first lockdown was lifted on most activities except a few like bars and sports activities.

### Study design and population

This was a mixed methods cross-sectional study using a sequential explanatory approach. Qualitative data was collected for triangulation and to complement findings from the quantitative study. The study participants included household heads of slum dwellers aged 18 years and above for the quantitative component, and purposively selected CHWs and community members for the qualitative part. In the qualitative component of the study, we used focus group discussions (FGDs) and photovoice. Photovoice is a visual qualitative method used in community-based participatory research to document insights and perspectives which raise awareness of hidden or overlooked issues and aspects of the community [19].

### Sample size and sampling procedure

A total of 425 respondents were randomly selected for the face-to-face quantitative interviews using systematic sampling. This sample size was calculated using the Kish Leslie (1964) formula for cross sectional studies and the following assumptions were considered; a conservative proportion of 50% since no similar study had been done at the time, confidence level of 95%, power of 80%, and a non-response rate of 10%.

Systematic sampling was used to obtain respondents for the study. In each zone, we listed all the households with the help of the local chairperson and determined the ‘*k*th’ interval, *k* being the total number of households in the zone divided by the sample size of households needed in that zone. To select a starting point, we stood at the centre of the zone with guidance from the local chairperson and randomly selected the starting household using a table of random numbers. Subsequent households were selected using the “*k*th” number. In case the “*k*th” household selected was closed on the day of data collection or if there was no proxy respondent, we rescheduled the interview for the following day and if on two subsequent times there was no respondent, we considered the next house in the same direction.

Six FGDs were conducted with purposively selected participants based on those who are more knowledgeable and well versed with community wellbeing such as local leaders and elders.

For the photovoice method, we selected eight CHWs as the photographers—four CHWs (two females and two males) were identified in each of the two study areas. The CHWs were identified with the help of other CHWs and the local political leaders in the study areas. Selection of the CHWs was based on their area of jurisdiction, level of education, occupation and marital status to ensure diverse representation and capturing of rich photographs from their areas of influence [20].

### Data collection

Interviewer administered face-to-face interviews were conducted with household heads using a semi-structured questionnaire in the local language, Luganda, to assess the unintended socio-economic and health consequences due to the COVID-19 pandemic and response activities. Impact on the various socio-economic and health factors was assessed using a pre-determined scale of high, moderate, low and no impact adapted from an impact classification proposed by Lavanya, N. & Malarvizhi, T. (2008) [21]. High impact translates to a rating between 80 and 100%, moderate 30 to 79%, low 1 to 29% and no impact as 0%. The pre-determined scale was readout to the respondents to rate themselves on the impact experienced. Eight research assistants with research experience in quantitative data collection were recruited and trained on data collection. The questionnaire was uploaded on mobile phones via the Kobocollect toolbox application for data collection.

We also conducted six FGDs in Luganda, the local language, to further assess community socio-economic and health consequences due to COVID-19 and response activities. By the 6th FGD, there was no more new information arising from the interviews. The FGDs were conducted within 50 min to 1 h with 7–8 participants per session using a guide. Two FGDs included only male participants; two had only female participants while two had both male and female participants. Each FGD was moderated by a researcher experienced in conducting FGDs, key notes taken, and the discussion audio recorded.

For photovoice, we used photographs to explore the immediate socio-economic and health consequences due to COVID-19 and response activities. An initial meeting was held between the study team and the selected CHWs to introduce the study to them. Using a guide*,* the selected CHWs were trained in data collection using photography in a 6 h workshop. The training covered study objectives, ethics in photography, use and care of cameras, professionalism and obtaining informed consent from community members before taking the photographs. The training on ethics was guided by findings from a study which explored ethical considerations in the work of a CHW [22]. In the workshop, pre-determined themes on the socio-economic and health consequences due to COVID-19 and response activities were discussed to ensure all the eight CHWs had the same understanding of what photo moments to look out for.

The CHWs took as many photos as possible in 1 month using the cameras given to them by the study team to capture situations in their communities that were related to socio-economic and health consequences arising from the COVID-19 pandemic and response activities. A field guide with pre-determined themes was used to guide the photography. Photographers were however at liberty to take photos related to the study even if they did not fall under any pre-determined theme. The research team conducted onsite supervision during photography to review the photos to ensure that they were appropriate and clear. Emerging challenges in the field were also discussed and addressed during the supervision. Four (4) weekly meetings lasting approximately 3–4 h were held every Saturday with CHWs and the research team to discuss the photos taken during the week. The number of weekly meetings was dependant on theoretical content saturation. In the weekly meetings, each CHW was asked to talk about their photos and discuss how the pictures related to their lives, those of the community, and the pre-determined themes of the study. In case CHWs were unable to take a photo either because of no consent given or unfavourable conditions, they took notes explaining the circumstances noted. The notes were discussed alongside the photos in the weekly meetings. The meetings were moderated by a researcher with experience in using photovoice, and notes of emerging issues taken by a note taker. All discussions for the meetings were audio-recorded.

### Quality control and assurance

All study tools were pretested in a similar setting, Bwaise II slum, one of the zones neighbouring the two study sites in Kawempe division to test the reliability and validity of the questions and to inform the data collection process. Interviewers were trained on pretesting the tools during the general training on data collection. During pretesting, each of the eight research assistants interviewed four household heads giving a total of 32 interviews. During the interviews, notes were taken for emerging problems in the interpretation, wording and flow of questions. In addition, one focus group discussion was conducted and emerging issues noted down. Sample size for pretesting was informed by previous studies that recommend a number between 30 and 75 to be able to capture about 75% of all high impact problems [23, 24]. Following pretesting, the research team met with the research assistants to discuss the emerging issues and how to improve design of the tools. The tools were revised prior to data collection to ensure they yielded the information required. Meetings were also held at the end of each day to check for consistency, completeness, and to ensure proper data collection. The study tool on the phone was fitted with checks to ensure data completeness and accuracy.

### Data management and analysis

All data were stored in password protected computers with no personal identifiers. Data from Kobocollect was downloaded from the app into an excel file, cleaned and imported into Stata version 14.0 for further cleaning and analysis. Quantitative data were analysed descriptively generating frequencies and proportions. To measure the association between socio-demographics and limited access to food during the COVID-19 pandemic, we ran a modified Poisson regression via generalized linear models to obtain prevalence ratios (PRs). Prevalence ratios were most preferred over odds ratios because the proportion of our outcome variable was > 10%, which would have given biased estimates [25]. Variables that had *p* values of up to 0.2 and those known from literature to be associated with limited access to food during disease outbreaks were included in the multivariable model. All inferential statistics were achieved at 95% confidence interval and 5% alpha level.

Audio recordings from the FGDs and photovoice meetings were transcribed verbatim in their original language of recording and translated to English if they were conducted in the local language. Three researchers read through the transcripts several times to familiarise themselves with the data after which they developed a codebook. This was followed with line by line coding by the three independent people. The independent lists of codes from the three researchers were reviewed by two core study team members to assess intercoder agreement. Any discrepancies were clarified and resolved by comparing each coder’s results with raw data until consensus was reached. Coded transcripts were then uploaded into the qualitative analysis software ATLAS.ti Version 7 for thematic analysis using the deductive and inductive approaches. Quotes were then selected to represent the main themes emerging from the study.

For the photograph analysis, at the end of the 1 month, all photos taken in the study and summaries of the main findings from each weekly meeting were presented back to the CHWs in a meeting. The CHWs were then asked to identify new themes arising beyond the pre-determined themes. The CHWs grouped photos per theme and also identified and selected those that best represented each theme and their community through consensus.

### Dissemination and interpretation of study findings

Findings from the study were disseminated in a workshop involving CHWs who participated in the study and others from the study area, community leaders, health workers, researchers and representatives from the Ministry of Health and Public Health Department of Kampala Capital City Authority. In the workshop, the selected photos for each theme were displayed for further interpretation and discussion. The photos were presented by the researchers while the photographers provided more contextual information about them which elicited discussion on what could be done to prevent re-occurrence of the same consequences in the event of another pandemic.

### Ethical considerations

We obtained ethical approval from the Makerere University School of Public Health Higher Degrees and Research Ethics Committee *(HDREC No.877)* and the Uganda National Council of Science and Technology *(registration number SS638ES)*. Written informed consent was obtained from all study participants before data collection. No photographs identifying an individual were used without the written consent of both the photographer and the identified person. The study team was trained to adhere to the COVID-19 prevention measures during field engagements and constantly supervised for compliance.

## Results

### Socio-demographic characteristics of the respondents

Out of the 425 respondents for the face-to-face interviews, most were females (66.4%; 282/425); aged 26 to 33 years (26.8%; 114/425); had attained secondary as their highest level of education (46.6%; 198/425); and were Muslims (32%; 136/425). Sixty three percent (268/425) of the respondents reported earning an average of between 50 and 300,000 Ugandan shillings (less than 100 dollars) per month *(*Table 1*).*

**Table 1:** Socio-demographic characteristics of the respondents

Variable	Frequency (***n*** = 425)	Percentage (%)
Sex		
Male	143	33.65
Female	282	66.35
Age (years)		
18 to 25	78	18.35
26 to 33	114	26.82
34 to 41	96	22.59
42 to 49	65	15.29
≥ 50	72	16.94
Mean (SD)	37.22 ± 12.6	
Highest level of education		
No formal education	42	9.88
Primary	155	36.47
Secondary	198	46.59
Tertiary	30	7.06
Marital status		
Married	166	39.06
Single	94	22.12
Co-habiting	76	17.88
Divorced / separated	59	13.88
Widowed	30	7.06
Religion		
Anglican	131	30.82
Catholic	99	23.29
Muslim	136	32
Seventh Day Adventist	8	1.88
Pentecostal	41	9.65
Others	10	2.35
Occupation		
Casual labourer	46	10.82
Employed	44	10.35
Self-employed	221	52
None	113	26.59
Others	1	0.24
Average income earned per month (Ugandan shilling)		
50–300,000/=	268	63.06
300,001- 500,000/=	41	9.65
500,001-1,000,000/=	2	0.47
None	114	26.82

### Socio-economic and health consequences due to COVID-19 and response activities

Most respondents reported: limited access to food (71.1%; 302/425); disruption in education (77.1%; 270/350); a drop in daily income and wages (86.1%; 329/382); loss of employment, (63.1; 125/198); limited access to cooking energy (52.7%; 224/425); and limited access to transport (68.5%; 291/425). Most respondents mentioned no impact of COVID-19 on: access to water (65.9%; 280/425); sanitation and hygiene facilities (68.7%; 292/425); and access to health care services (71.5%; 304/425). Twenty five percent of the respondents 25.4%; 86/338) reported domestic violence. Almost half (40.2%; 171/425) of the respondents reported mental health challenges including reduced sleep, anxiety and urge to drug use (Table 2*)*.

**Table 2:** Immediate socio-economic and health consequences due to COVID-19 in Bwaise I and Bwaise III, Kampala Uganda

Impact on:	High	Moderate	Low	No impact
Household access to food	302 (71.06)	41 (9.65)	20 (4.71)	62 (14.59)
Household access to water	96 (22.59)	28 (6.59)	21 (4.94)	280 (65.88)
Household access to sanitation and hygiene services	82 (19.29)	31 (7.29)	20 (4.71)	292 (68.71)
Education (*n* = 350)	270 (77.14)	9 (2.57)	4 (1.14)	67 (19.14)
Household daily wages and incomes (*n* = 382)	329 (86.13)	21 (5.50)	4 (1.05)	28 (7.33)
Employment (*n* = 198)	125 (63.13)	10 (5.05)	8 (4.04)	55 (27.78)
Domestic violence (*n* = 338)	86 (25.44)	22 (6.51)	21 (6.21)	209 (61.83)
Social behaviours	40 (9.41)	8 (1.88)	7 (1.65)	370 (87.06)
Family welfare	118 (27.76)	30 (7.06)	25 (5.88)	252 (59.29)
Household lighting	136 (32.00)	32 (7.53)	26 (6.12)	231 (54.35)
Household cooking energy	224 (52.71)	34 (8.0)	17 (4.00)	150 (35.29)
Transport	291 (68.47)	22 (5.18)	22 (5.18)	90 (21.18)
Health	87 (20.47)	43 (10.12)	17 (4.00)	278 (65.41)
Access to health care	89 (20.94)	19 (4.47)	13 (3.06)	304 (71.53)
Mental health	171 (40.24)	27 (6.35)	23 (5.41)	204 (48.00)

### Factors associated with one of the highest reported impact on socio-economic and health vulnerabilities due to COVID-19 in Bwaise I and Bwaise III, in Kampala Uganda

#### Impact on household access to food

After adjusting for potential confounders, respondents aged 25 to 33 years (adjusted PR = 1.19, 95% CI: 1.01–1.41); 42 to 49 years (adjusted PR = 1.22, 95% CI: 1.01–1.47); and ≥ 50 (adjusted PR = 1.22, 95% CI: 1.01–1.48) were more likely to have limited access to food. Respondents who reported earning an average monthly income of 300,001/= and above were 0.74 times less likely to have limited access to food compared to those with no monthly income (adjusted PR = 0.74, 95% CI: 0.55–0.98), see Table 3.

**Table 3:** Crude and adjusted factors associated with the impact of COVID-19 on household access to food and socio-demographic characteristics

Attributes	Impact of COVID-19 on household access to food		Unadjusted PR (95% CI)	Adjusted PR (95% CI)	***P*** value
	Low (***n*** = 82)	High (***n*** = 343)			***P*** value
Sex					
Male	29 (20.3)	114 (79.7)	0.98 (0.89–1.09)	1.03 (0.93–1.15)	0.542
Female	53 (18.8)	229 (81.2)	1	1	
Age (years)					
18 to 25	23 (29.5)	55 (70.5)	1	1	
26 to 33	19 (16.7)	95 (83.3)	1.18 (1.01–1.39) *	**1.19 (1.01–1.41)**	**0.044**
34 to 41	17 (17.7)	79 (82.3)	1.16 (0.98–1.38)	1.18 (0.99–1.41)	0.063
42 to 49	10 (15.4)	55 (84.6)	1.20 (1.01–1.43) *	**1.22 (1.01–1.47)**	**0.038**
≥ 50	13 (18.1)	59 (81.9)	1.16 (0.97–1.39)	**1.22 (1.01–1.48)**	**0.037**
Highest level of education					
No formal education	7 (16.7)	35 (83.3)	1	1	
Primary	20 (12.9)	135 (87.1)	1.05 (0.90–1.21)	1.05 (0.91–1.21)	0.496
Secondary	45 (22.7)	153 (77.3)	0.93 (0.79–1.08)	0.96 (0.82–1.12)	0.596
Tertiary	10 (33.3)	20 (66.7)	0.80 (0.60–1.07)	0.84 (0.61–1.15)	0.270
Marital status					
Married	42 (17.4)	200 (82.6)	1	1	
Single	22 (23.4)	72 (76.6)	0.93 (0.82–1.05)	0.99 (0.87–1.12)	0.868
Divorced / separated	12 (20.3)	47 (79.7)	0.96 (0.84–1.11)	0.93 (0.81–1.07)	0.496
Widowed	6 (20.0)	24 (80.0)	0.97 (0.80–1.17)	0.89 (0.74–1.07)	0.225
Occupation before pandemic					
Casual labourer	7 (15.2)	39 (84.8)	**1**	1	
Employed	13 (29.6)	31 (70.4)	0.83 (0.66–1.04)	0.88 (0.70–1.11)	0.277
Self-employed	44 (19.8)	178 (80.2)	0.95 (0.82–1.09)	0.97 (0.84–1.12)	0.700
None	18 (15.9)	95 (84.1)	0.99 (0.86–1.15)	0.88 (0.72–1.08)	0.225
Average monthly income					
None	16 (14.0)	98 (86.0)	1	1	
50–300,000/=	53 (19.8)	215 (80.2)	0.93 (0.85–1.03)	0.84 (0.69–1.01)	0.060
300,001- above	13 (30.2)	30 (69.8)	0.81 (0.66–1.00)	**0.74 (0.55–0.98)**	**0.035**

From the analysis of FGDs and photovoice findings, the following themes emerged under socio-economic and health consequences due to COVID-19 and response activities; limited access to food; negative impact on children rights and education; poor housing and lack of accommodation; negative social behaviours; negative impact on family and child care; reduced income and employment; and negative impact on health and access to health care services.

#### Negative impact on children rights and education

Closure of schools due to COVID-19 with no clear mechanism of continued learning at home exposed most children to several consequences. Photovoice participants noted that some children were exposed to forced labour either to provide extra income to the family or as their own initiative. Engagement of children in labour was mentioned by participants as likely to expose them to COVID-19, sexual violence, early pregnancies, undesirable behaviours and increase school dropout. A photographer explained the extent of the impact on children rights and education:

*“Many children have become involved in petty trade and most of these are unwilling to return to school. I am sure a substantial percentage of these children will drop out of school when schools fully reopen. Sending children especially girls to sell merchandise is problematic because they may get molested or sexually assaulted by the men they find along the way”* (Photographer 3, female, age 38)

**Fig. 1:**
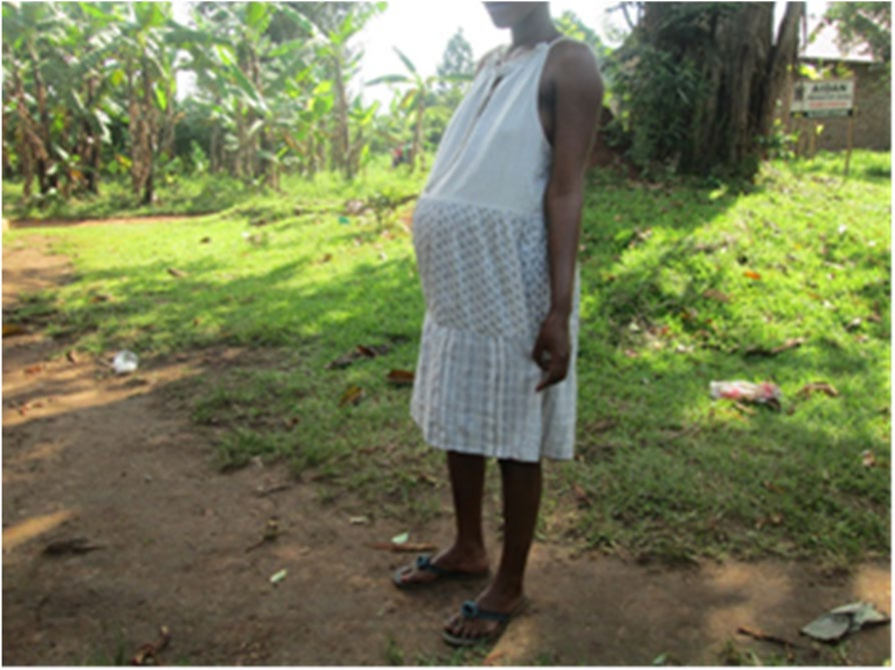
A 14-year old school going child who got pregnant during the lockdown

Participants in both the FGDs and photovoice mentioned increased teenage pregnancies and early child marriages since the onset of COVID-19 (Fig. 1). This was attributed to teenage girls being out of school and redundant at home with their parents and guardians focused on making income for survival. Unwanted pregnancies were also attributed to the girls lacking basic needs hence looking out to men for survival.

*“My issue is that since schools were closed during COVID-19 outbreak, we saw many girls drop out of school. These girls have got unwanted pregnancies and as a mother, I can’t take care of the pregnant girl and the unborn baby because I have no money or a job. I could have taken care of the pregnant girl but the workplaces were closed. We have been affected so much by this situation.”* (Female FGD, Participant 2, Bwaise III)

#### Limited access to food

**Fig. 2:**
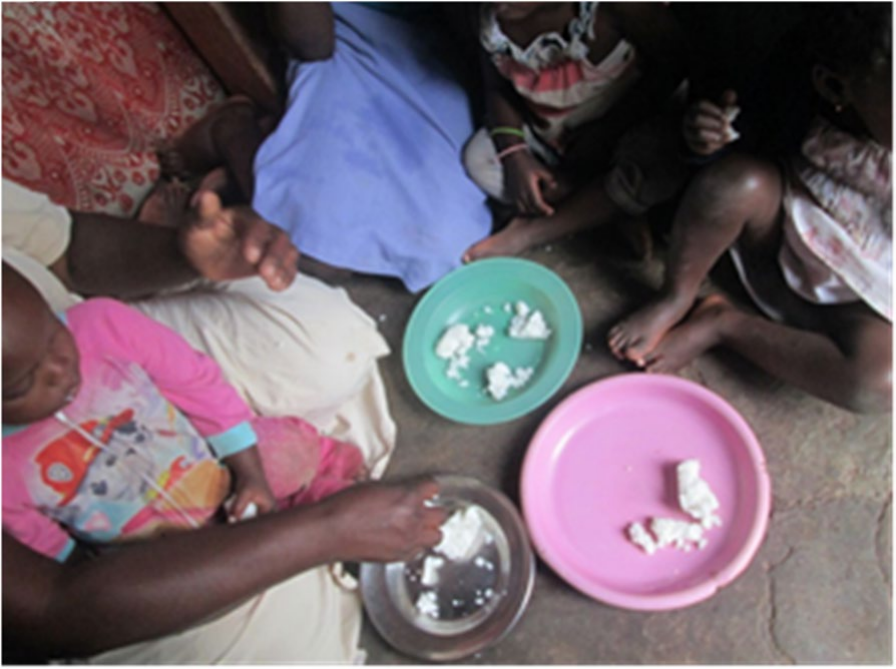
A family eating reduced food portions

Many study participants in the FGDs and photovoice mentioned reduced access to food given that most had their income reduced, lost jobs and some had their businesses shut down during the pandemic. Reduced access to food was expressed in terms of no money to buy the food, increased food prices and limited availability of certain foods. Patients suffering from chronic illnesses were affected more because they had to take their medication on an empty stomach. Most participants mentioned reduced: frequency of meals, eating of a balanced diet and portions (Fig. 2) as a coping strategy.

*“…COVID-19 affected me so much because I was used to eating two meals a day but I can no longer do that. We now have one meal a day and that is supper. We take a cup of tea a day at 4pm and prepare the little food we have. At 7pm, we have our supper and sleep. The earnings also reduced.”* (Female FGD, Participant 8, Bwaise III)

#### Impact on family and child care

Cases of domestic violence in the slum communities were mentioned to have increased during the COVID-19 pandemic. Participants attributed this to no money to sustain homes, increased leisure time at home and loss of jobs by the bread winners. Relatedly, some families were said to have broken up due to the heightened violence and also increased demands for basic needs from the family.

*“… I might even shed a tear while talking. First of all, i was beaten up during that time (lockdown). I was beaten up because I had no money. The child would ask me for food but I had nothing. My husband would ask for food and later beat me up. There is nothing I gained from the situation except being beaten up.”* (Female FGD, Participant 4, Bwaise III)

#### Increased drug use and abuse

**Fig. 3:**
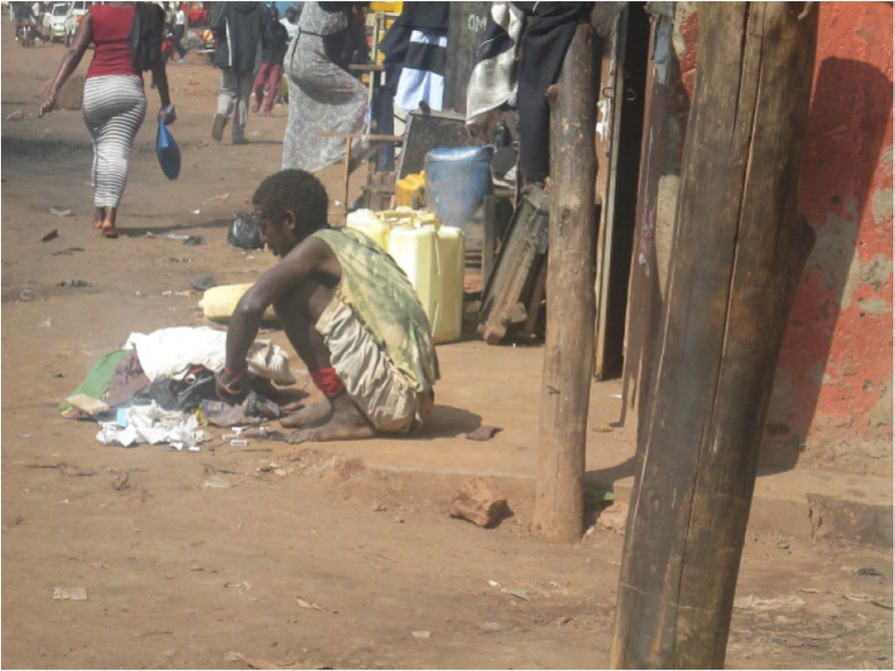
A new drug addict now gone mad

Study participants in the photovoice also noted an increase in the number of people using drugs since the beginning of the lockdown (Fig. 3). It was reported that most people are cited drinking alcohol from early morning hours to evening and others smoking cannabis and marijuana, with some developing mental illnesses. Some photovoice participants attributed the increase in drug use to increased stress in the population due to loss of jobs and income and others attributed it to several people having too much time and idleness, most of them having lost jobs and businesses.

*“…the truth is we have had a rising number of such cases (cases of drug abusers) due to COVID-19 situation. Because, they (community members) searched for what to do and failed to get a job. They looked for money and what to eat and failed to get so they got frustrated and started drugs. That was a sensible person* (Fig 5) *before COVID-19 situation but now, see what he has become.”* (Photographer 7, male, age 32)

#### Unintended immediate economic consequences due to COVID-19

##### Reduced income and loss of employment

Most participants in photovoice and FGD mentioned loss of employment as one of the consequences of COVID-19. The loss of employment was largely a result of most companies laying off staff and others because of the shutdown of businesses during the lockdown. Loss of employment was said to have greatly impacted access to basic needs and family stability.

*“…people lost jobs, curfew also affected food sellers because customers were limited, those with bars sent away many workers who are now jobless; those who have lodges aren’t functional yet it also employed many people; boda-boda riders were affected by curfew as well, schools were also affected so much.”* (Male FGD, Participant 1, Bwaise III)

During the lockdown and after, several businesses in the community collapsed and others had reduced performance in terms of customers and income generation. Collapse of businesses was attributed to some people using up the capital for food to survive during the lockdown and reduced performance attributed to most people having no money to buy from the business vendors.

*“COVID-19 situation affected me so much because I used to have a retail shop and the school going students would buy from me. I am a widow with many children but I had to close the shop. The little capital I had was used to buy and stock food during the COVID-19 lock down and every day would just pass by. I even lacked money to buy water and sugar.”* (Mixed FGD, Participant 6, Bwaise III)

##### Inability to afford housing and accommodation

**Fig. 4:**
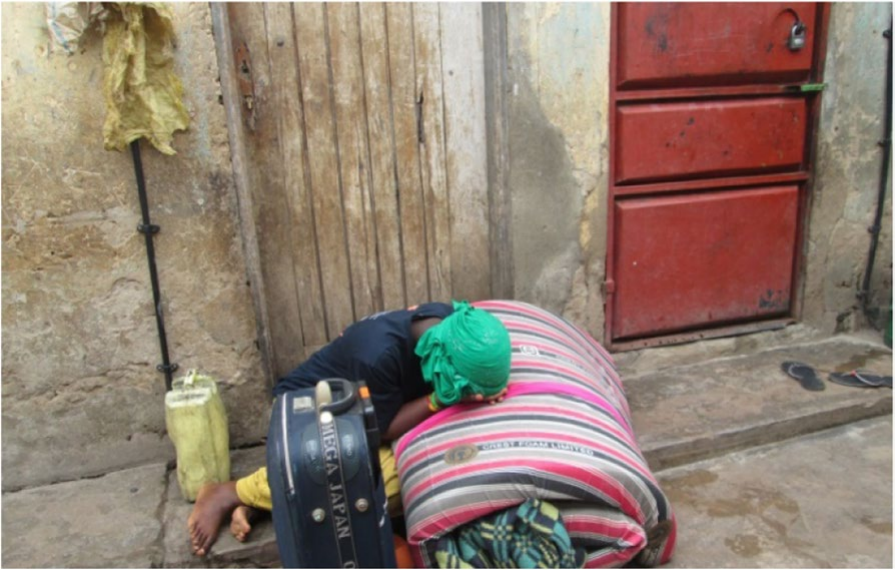
A slum resident after being evicted from her rental house by the land lord

Both photovoice and FGD participants mentioned several community members have since the start of COVID-19 accumulated rent arrears with some being evicted from the houses and shops (Fig. 4). Accumulated rent arrears came because of unemployment and reduced income during the lockdown. Some community members were said to have resorted to residing in animal houses offered by neighbours.

*“This photo shows the effect of COVID-19 on housing. The property in the photo belonged to a tenant who had been evicted. The tenant had rent arrears of four months and the landlord could not tolerate him any longer. Therefore, COVID has left many people homeless.”* (Photographer 4, male, age 64)

#### Unintended immediate health consequences due to COVID-19 and response activities

##### Reduced health and limited to access health care services

**Fig. 5:**
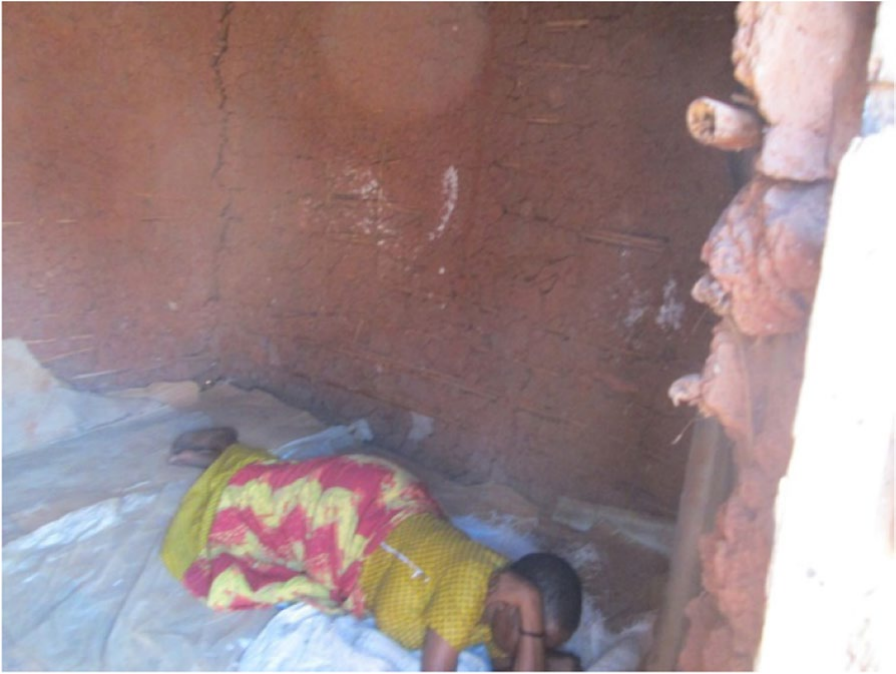
A community member who had been sick for days lying helpless in her house with no medical care

Due to limited movements during the lockdown, increased transport fares and no income, some slum dwellers resorted to not seeking health care when sick. They stayed indoors hoping to get better (Fig. 5). More so, most said they were ignored and did not receive care when they visited the health centers as all attention was on COVID-19 patients. Those on chronic medication like people living with HIV, could not refill their medicines because of transport challenges.

*“Some people who had chronic illnesses such as diabetes, HIV/AIDS and hypertension either worsened or died because there was very limited access to routine healthcare services. The limited access to health care services was due to absence of transport means to health facilities and change in priority of healthcare services to mainly focus on COVID-19 patients and neglecting other diseases.”*(Photographer 5, female, age 60)

Respondents in the FGDs reported increased mental health conditions because of COVID-19 restrictions and related challenges. Most mentioned increased stress as a result of many needs against no money; others mentioned increased anxiety and disrupted sleep.

*“We have all lost our minds during this situation. You find people walking while talking to themselves on the street. Others have tried sleeping but failed and others have got accidents because they walk without thinking. The landlords evict us and when you explain to them they also claim they built houses to make money. The children ask for food and when you talk to your husband, he just walks away. What I think is that the mental health issues will worsen in future because of this situation. People will only think 50% because of this stress.”* (Female FGD, Participant 8, Bwaise III)

## Discussion

This study assessed the unintended socio-economic and health consequences due to COVID-19 and the response activities among slum dwellers in Kampala, Uganda. Most respondents reported limited access to food, education, daily income, wages and employment, housing, mental health challenges, and increased domestic violence. Other challenges included effects on children rights and education. The unintended consequences due to COVID-19 reported in this study include critical determinants of health and pose a major threat to the wellbeing and health of these communities which could also increase their vulnerability to COVID-19.

Our findings revealed challenges in access to food both in terms of physical and financial access. Most respondents accessed only one meal a day, reduced portions or were not able to have a balanced diet. Slum dwellers largely depend on daily wages for food and other essential requirements [26, 27]. The prolonged lockdown without socioeconomic support therefore exposed them to severe challenges related to access to food. Uganda integrated food distribution for vulnerable urban populations to reduce the likely impact but this was not adequate in quantities and could not be sustained long enough to avert the challenges with access to food [15]. Our findings are similar to findings in other studies in Uganda and elsewhere where food security outcomes were worse among poor households which depend on labour income [28, 29]. Limited access to food could lead to reduced nutrient intake consequently leading to diet-related and nutrition-related diseases [30]. Nutrition-related diseases are often associated with reduced immunity which will likely expose more people to COVID-19 and other infections [31].

The negative impacts on children’s rights and education arose among the challenges. COVID-19 related restrictions led to disruption of education which is likely to have long lasting impact on the children including school dropout, teenage pregnancies and early marriages for girls, with some unlikely to go back to school due to lack of school fees. Returning to school after giving birth is not guaranteed because of the stigma that could arise but also increased responsibility to take care of the child [32, 33]. In northern and eastern Uganda, an increase in cases of young girls forced to sell sex in return for cash, food, or even sanitary products during the COVID-19 pandemic was reported [34]. Teenage pregnancies also increased by 17% as reported in the Health Management Information System (HMIS) national database and Uganda Child-Helpline during the pandemic [35]. In the Ebola outbreak in West Africa, similar consequences were observed such as teenage pregnancies, school drop outs and early marriages for school going children [36, 37]. Children are vulnerable to social and health effects hence there is need to protect them from things that threaten their well being [38–41].

A reduction in daily income, wages and employment was reported. Due to the lockdown, most businesses and formal workplaces were unable to operate leading to no income generation and downsizing. Studies elsewhere have reported financial insecurity, loss of employment and reduced income due to COVID-19 related lockdowns [8, 15, 34, 42–44]. Reduced income affects health care seeking, limited access to basic needs like food and water and increased crime rate [45, 46]. In desperation and pursuit of income for survival, people are also likely to engage in risky activities like congregating in large numbers and shunning of the recommended prevention guidelines which may lead to their exposure to COVID-19 [47]. Therefore, the economic and financial wellbeing of vulnerable populations should be catered for as part and parcel of the pandemic response strategies.

Domestic violence was reported to have increased during the COVID-19 pandemic. Increased domestic violence could have been as a result of increased financial stress in families, increased time of closed stay and unfulfilled expectations from partners. Studies elsewhere reported some people losing their lives as a result of the domestic violence, and some sustaining bruises from beatings during the COVID-19 pandemic [34, 48, 49]. In Peru, calls of domestic violence on women to the national helpline increased by 56% in April 2020 [49]. In Africa, gender based violence increased during the COVID-19 pandemic from as low as 10% in Mali to as high as 50% in Liberia [50, 51]. Relatedly, the Ebola outbreak in West Africa also resulted in increased cases of gender based violence among teenage girls and young women [37]. Increased domestic violence threatens the wellbeing of society and family.

Our qualitative findings revealed limited access to health care services and worsening of some health conditions. This was reported among persons with chronic illnesses like HIV/AIDS, diabetes, hypertension and others. Studies in other countries have reported reduced health care utilisation and disrupted health care services [52–55]. Reduced health care seeking may lead to poor health outcomes for other diseases and increased risk for community spread of COVID-19 and other infectious diseases due to cases not reporting to the health facilities. Strategies to strengthen and sustain other health services during pandemics such as COVID-19 are essential in ensuring good health outcomes across the board.

Mental health challenges were also prominent in this community. Increased mental health challenges such as anxiety, disruption in sleep patterns, stress and so forth may have resulted from forced stay at home during the lockdown, separation from loved ones, restricted movements, uncertainty, boredom and fear of infection. Mental health challenges especially psychological effects have been reported elsewhere [15, 54, 56–59]. Disease pandemics are inherently stressful hence adding other stressors such as restriction in movements, work and separation from loved ones worsen the situation, and could have longer term effects after the pandemic. Mental health challenges could also arise from the stigma that is meted on the survivors of infectious disease such as COVID-19 and their families. However, mental health is not always prioritised in disease response strategies. Pandemic response strategies should therefore be integrated with psychosocial and mental health interventions.

Whereas the study found no impact on access to water, sanitation and hygiene (WASH) services during the COVID-19 pandemic, attention still needs to be given to this area to ensure continued provision of these services in the informal settlements. Similarly, a study in India found no impact on access to sanitation and hygiene services except for water supply [60]. No impact on access to WASH services could have been because of the emphasis and investments into these services by the government and individuals since they are currently seen as a major solution to interrupt transmission of COVID-19. Informal settlements have for long been known to have limited access to WASH services due to design of the settings and low economic abilities of the dwellers to pay for services [27, 61–63]. Limited access to WASH services may lead to disease outbreaks such as cholera, diarrhoea and typhoid resulting into other epidemics further challenging the already constrained health system in the country. Furthermore, WASH services are essential in the fight against COVID-19 hence the need to strengthen their provision and make them accessible to vulnerable communities [63, 64]. Increased impact on other socio-economic and health consequences of COVID-19 could also result into coping mechanisms that may affect availability of WASH services at the household level.

A strength to our study is the use of mixed methods to triangulate the unintended socio-economic and health consequences due to COVID-19 in this vulnerable population. We used the photovoice method which enables community participation in the process and raises awareness of hidden or overlooked issues in the community. By the end of the study, the photographers, who were also CHWs, attested to the fact that the photovoice method made them more knowledgeable about issues that arose in their communities during the COVID-19 pandemic. In addition, the study expanded their networks both in the community and with the researchers and policy makers which was an opportunity to learn more and improve their work in the community. A study which used the photovoice method to explore community level barriers affecting maternal health in a rural district had similar findings and underscores the need for continued use of participatory approaches in research [65].

Our study did not establish the baseline status to objectively demonstrate the changes due to COVID-19 and relied on self-reported impact which may be subject to social desirability bias. However, use of the photovoice method which is observational in nature limited possibilities of erroneous self-reported results. Further studies are recommended to establish the extent of impact of the COVID-19 pandemic and mitigation interventions. Innovative methodologies are required to measure the extent of the impact amidst the on-going pandemic. We did not explore socio-economic and health consequences of COVID-19 by gender which could be assessed in future studies to understand the impact especially on women who were already vulnerable even before the pandemic. Research gaps also remain on coping mechanisms for the unintended consequences of COVID-19 some of which may be further escalating the effects or disease transmission and outcomes.

## Conclusion

The negative consequences of COVID-19 and related restrictions were quite severe among the vulnerable slum dwellers in Kampala. Most respondents reported a high impact on access to food, education, daily income and wages, employment, and access to cooking energy. Domestic violence and mental health as well as effects on child education and rights were also prominent. These findings emphasize the need for comprehensive preparedness and response strategies and plans that cater for the socioeconomic needs especially for the most vulnerable populations. During design of response and prevention activities for disease outbreaks, protocols should be established and mainstreamed to protect vulnerable groups in the community especially children and women. The impact on education and the young school going children could be long-term and requires both immediate and long-term mitigation strategies to ensure continuity of education and minimize future socioeconomic challenges.

## Data Availability

Data and materials supporting findings in this manuscript are available upon reasonable request through the Institutional Review Board of Makerere University School of Public Health. They can be contacted at hdrecadmin@musph.ac.ug.

## Abbreviations

CBD: Central Business District
CHWs: Community Health Workers
COVID-19: Coronavirus Disease
FGDs: Focus Group Discussions
HCD: Human-centred design
HDREC: Higher Degrees Research and Ethics Committee
HIV: Human Immunodeficiency Virus
IRB: Institutional Review Board
KCCA: Kampala Capital City Authority
KIIs: Key Informant Interviews
KIs: Key informants
NGOs: Non- Government Organisations
SDG: Sustainable Development Goal
SOPs: Standard Operating Procedures
USA: United States of America
WASH: Water Sanitation and Hygiene
WHO: World Health Organisation
